# Rye and Rye Bran as Components of Diets in Piglet Production—Effects on *Salmonella* Prevalence

**DOI:** 10.3390/ani13142262

**Published:** 2023-07-10

**Authors:** Christian Homann, Isabell Eckey, Bussarakam Chuppava, Klaus Teich, Juhle Buch, Andreas Zimmermann, Martin Kaltschmitt, Richard Grone, Volker Wilke, Christian Visscher

**Affiliations:** 1 Institute for Animal Nutrition, University of Veterinary Medicine, Foundation, 30559 Hannover, Germany; christian.homann@tiho-hannover.de (C.H.); sekretariat-tierernaehrung@tiho-hannover.de (I.E.); bussarakam.chuppava@tiho-hannover.de (B.C.); christian.visscher@tiho-hannover.de (C.V.); 2 SAN Group Biotech Germany GmbH, 49685 Emstek, Germany; teich@anicon.eu (K.T.); buch@anicon.eu (J.B.); 3 Institute of Environmental Technology and Energy Economics, Hamburg University of Technology, 21073 Hamburg, Germany; andreas.zimmermann@tuhh.de (A.Z.); kaltschmitt@tuhh.de (M.K.); 4 KWS Lochow GmbH, 29303 Bergen, Germany; richard.grone@kws.com

**Keywords:** *Salmonella*, pigs, sows, pre-harvest, foodborne pathogen, epidemiology, rye, rye bran, animal nutrition, livestock

## Abstract

**Simple Summary:**

Salmonellosis in humans is still a serious disease, which is mainly caused by the consumption of contaminated food. Functional ingredients in feed are expected to reduce susceptibility to the ubiquitous *Salmonella* and thus prevent food contamination. Rye is becoming increasingly popular in pig feeding. The use of by-products such as rye bran in pig feeding is also of great interest. Rye and its by-product rye bran offer the possibility of introducing high-quality feedstuffs with positive effects on the pig’s intestinal health into a feed. These effects are caused by functional ingredients that are metabolized by microorganisms in the large intestine. Previous studies have shown the positive effect of reducing *Salmonella* prevalence in young pigs. This study investigated in a field study whether rye or rye bran has a positive effect on *Salmonella* prevalence in gilts, sows, and piglets. The antibody titers of the rye groups in the gilts were significantly lower. This suggests that the use of rye leads to lower incidents of infection, but this effect was not reflected in the environmental samples. The inconsistent presence of *Salmonella* and the experimental design were not sufficient to show a clear effect in a field study.

**Abstract:**

The nutritional benefits of rye (and therefore rye bran) are mainly due to its high content of fermentable dietary fiber, the non-starch polysaccharides (NSP). Microorganisms in the large intestine are able to convert these into short-chain fatty acids (SCFA), including butyrate. Butyrate strengthens the epithelial barrier function in the colon by nourishing the enterocytes and inhibiting the spread of *Salmonella* in the intestinal tract. Therefore, the aim of this study was to test under field conditions whether a diet with rye or rye bran as the main ingredient for gilts, sows, and weaned piglets is associated with a lower *Salmonella* prevalence. Depending on the age groups, between 20–30% rye or between 15–20% rye bran was used in the experimental group. A total of *n* = 1983 boot swabs, *n* = 356 fecal samples, and *n* = 1909 serum samples were examined. The results of this study show that rye or rye bran at the levels used had no apparent effect on the number of positive *Salmonella* samples. However, the *Salmonella* OD values in the experimental groups were significantly lower than in the control group. This suggests that the use of rye leads to a lower incidence of infection, but this effect could not be proven from swabs.

## 1. Introduction

Food safety has become an increasingly important issue at national and international levels in recent years [1]. According to the Robert Koch Institute, salmonellosis is the second most common notifiable bacterial gastrointestinal disease in Germany [2]. In 2021, 60,050 cases of Salmonellosis occurred in the EU [1]. As salmonellosis is a classic food-borne infection, several measures have been taken to control the disease [1,3]. Following early efforts in the Scandinavian region measures to reduce *Salmonella* were taken, for example, in Germany in 2007 [3]. Since then, pig farms have been required to have their pigs tested for *Salmonella* at the slaughterhouse according to a sampling key [3]. The results of the *Salmonella* antibodies allow the classification of the farms in three categories. Categorization is based on the percentage of positive results in the sampled group. Classification is then made into Category I (≤20% positive samples), Category II (>20% and ≤40% positive samples), or Category III (>40% positive samples). In case of classification in Category III, the farmer, together with the supervising veterinarian, must take measures to reduce the prevalence of *Salmonella* in the farm. The measures are limited to key points such as cleaning and disinfection, management, rodent control, and reduction in possible entry points [3]. There are many different approaches for reducing *Salmonella* in pork production. For example, increased biosecurity measures [4], coarser feed structure [5], or a pH reduction in the feed [6]. It is important to note that salmonellosis on pig farms is a multifactorial problem [7]. Extensive research in the area of feeding is necessary to make use of a promising possibility to reduce *Salmonella* [7].

As illness from *Salmonella* remains prevalent in pig production [8], there has been increased interest in the search for alternative interventions besides biosecurity and vaccination. Rye has many advantages when grown on dry and nutrient-poor soils [9]. Selective breeding has also made rye less susceptible to ergot alkaloids, allowing it to be used again in breeding animals [10]. Rye is also a valuable cereal in terms of sustainability. It has been shown that a compound feed with high proportions of rye in combination with regional protein sources leads to a lower carbon footprint while maintaining the same performance [11]. Despite its similarity to wheat feeding, a rye-rich diet has an influence on the physicochemical properties of the digesta of pigs [12]. Rye leads to longer-lasting satiety in the animals due to the higher viscosity and delayed gastric emptying. This can be important when feeding, for example, pregnant sows [12]. Of particular interest is the high content of non-starch polysaccharides (NSP) in rye [13]. This NSP is not digestible in the small intestine of the pig and is thus degraded by microorganisms in the large intestine. This results in increased formation of SCFA (short-chain fatty acids). Butyrate as a salt or ester of butyric acid has various influences on the intestine [14,15,16,17]. On the one hand, it serves as an energy supplier for colon cells [18] and protects the intestinal epithelia [15], and on the other hand, it also reduces inflammatory reactions and has bacteriostatic properties [14]. Especially the last mentioned property can be transferred to *Salmonella*. Gantois, et al. [17] and Lawhon, et al. [19] showed already years ago that butyrate reduces the multiplicity and pathogenicity of *Salmonella*. Chuppava, et al. [20] showed that rye feeding leads to a significant reduction in *Salmonella* excretion in the feces of young pigs. It is now necessary to check whether the positive aspects of rye or rye bran as a by-product of this also work under field conditions in piglet production (gilts, sows, and piglet rearing) and lead to reduced *Salmonella* excretion.

Using by-products in the feeding of pigs offers the possibility of nutrient utilization of these [21]. The food industry, in particular, offers high-quality components with high economic value [22]. Since bran represents about 10–15% of the grain weight [23], there is high waste in the milling process. Rye bran is rich in dietary fiber, vitamins, minerals, and other bioactive substances [24]. Since rye has many dietary beneficial aspects as mentioned above, its by-products are also of interest. Rye bran in particular contains many of the NSPs that can be converted to SCFA in the large intestine. Therefore, the use of rye bran is being investigated as a solution to reduce *Salmonella* in pigs.

Latently infected animals are of great importance for the introduction and spread of *Salmonella* in pig herds [25]. Thus, *Salmonella* control measures should not be limited to the individual animal, but to the entire herd. As *Salmonella* is present in the entire production chain, from piglets to fattening pigs, the reduction must already start with the piglet producer [7,26]. As the purchase of gilts in particular is a risk factor for the introduction of *Salmonella* onto the farm, there is great potential for improvement at the time of gilt integration or gilt quarantine [27]. The aim of this study is to show the effect of rye and rye bran on *Salmonella* prevalence in piglet production. A dietary approach with rye or rye bran for *Salmonella* could be applied in almost all pig production areas. Furthermore, rye, and rye bran in particular offer the possibility of substituting other feed grains, which is an economic incentive in addition to the potential impact on animal health.

## 2. Materials and Methods

### 2.1. Ethical Statement

The Ethics Committee for Animal Experiments of LAVES and LALLF (Lower Saxony State Office for Consumer Protection: reference 33.8-42502-05-20A557 and State Office for Agriculture, Food Safety and Fisheries Mecklenburg–Vorpommern: reference 7221.3-2-018/20) approved the animal experiments. The data were collected as part of the Rye-SaFe project (2813IP026), which is funded by the German Federal Ministry of Food and Agriculture.

### 2.2. The Farms and Animals

The study took place on three piglet farms (A, B, and C) in northern Germany from January 2021 to May 2023. Participation in the study was voluntary due to recurrent *Salmonella* infections. The farms were of different sizes (Farm A: *n*= 1000 sows, B: *n*= 230, and C: *n* = 1850). In all farms, the gilts integration, farrowing unit, and piglet rearing unit were examined. Farm A was a pure piglet producer with an attached piglet-rearing unit. Farm B was a closed system. In Farm C, 50% of the piglets were reared in the farm-owned piglet rearing unit. Farms A and B remount the sow herd with purchased gilts, while Farm C remounts from its own stock. On Farm A, the gilts were vaccinated in quarantine due to ongoing *Salmonella* problems. During integration, the gilts were vaccinated twice against *Salmonella* with an attenuated *S. typhimurium* vaccine strain (Salmoporc^®^, Ceva Tiergesundheit GmbH, Düsseldorf, Germany); the first dose was administered subcutaneously two weeks after arrival and the second dose four weeks later. During the rearing period, the piglets were kept for 7.5 weeks on Farms A and B, while on Farm C the piglets were rehoused in the middle of the period so that the piglets spent 3.5 weeks two times, i.e., 7 weeks, in rearing.

### 2.3. Diets

The first part of the study took place between January 2021 and July 2022 and focused on the influence of rye on *Salmonella* prevalence. The second part took place from March 2022 to May 2023 and focused on rye bran as a compound feed ingredient. As different age groups of pigs (gilts, sows, and piglets) have different nutritional needs, a different diet was developed for each age group. The experimental diets were always based on commercial farm diets, which were then converted to the same nitrogen and energy content including the required amount of rye or rye bran. Nutrient composition of all compound feeds can be seen in Table S1–S4. The amounts of rye and rye bran used in the experimental diets are shown in Table 1. As the farms required different experimental diets, these varied slightly in composition but always contained the prescribed amount of rye or rye bran. The form of the diet also varies among the farms. The feed in Farms A and B is in meal form and Farm C feeds with a liquid feed.

The experimental and control diets were routinely analyzed during the study. The Weender analysis was used to evaluate the raw nutrients in the diets used according to LUFA guidelines [28]. The starch and mineral content of the diets were also determined. The starch content and the results of the Weender analysis were used to calculate the energy content of the diets. High-performance chromatography (HPLC) was used for mycotoxin analysis to determine the important toxins ergotamine, deoxynivalenol, and zearalenone. One of the 12 ergot alkaloids, ergotamine, was used as a marker for the ergot alkaloids. In this way, the risk of contamination with mycotoxins or undesirable components could be detected and, if necessary, reduced.

#### 2.3.1. Gilt Diets

Two isonitrogenous diets were prepared for each of the experimental and control gilts. In the first part of the study, the influence of rye on *Salmonella* was investigated. For this purpose, an isonitrogenous and an isoenergetic diet containing 30% rye was developed based on the control feed of the farm (Table 2).

In the second part of the study, the influence of rye bran on *Salmonella* was tested. For this purpose, a diet with 20% rye bran was created (Table 3).

Since the rye bran trials could only be conducted in Farm A, an experimental diet for gilts was prepared only for this farm.

#### 2.3.2. Peripartum Diets

Isonitrogenous diets were also created in the peripartum period. In the first part of the study, 30% rye was used in the compound feed. In the second part of the study, 15% rye bran was added to the compound feed in order to achieve a sufficient energy supply for the lactating sows with high fiber content. The feeds are shown in Table 4 and Table 5.

In the second part of the trial, the feed had to be changed due to price increases (caused by the beginning of the war in Ukraine) after the second run, so two different experimental and control feeds with different energy densities (13.4 MJ ME and 13.2 MJ ME) were used. However, 15% rye bran was used in both experimental diets (Table 5).

#### 2.3.3. Piglet Rearing Unit

Piglet rearing was only investigated in the first part of the study. Here, 25% rye was used in the diets (Table 6). In order not to harm the optimal growth of the weaned piglets, the experimental diets were fed after a certain time in the flat deck pen. On Farms A and B, piglet-rearing diets were carried out in two phases so the experimental diet was only used in the second piglet-rearing diet. The piglet-rearing diet on Farm C was fed in four phases. Piglet-rearing diets three and four were used as control diets and then isonitrogenically and isoenergetically reformulated into experimental diets. The farm-specific diet without rye was used as a control diet on all farms.

### 2.4. Experimental Design

#### 2.4.1. Gilt Integration

**Figure 1:**
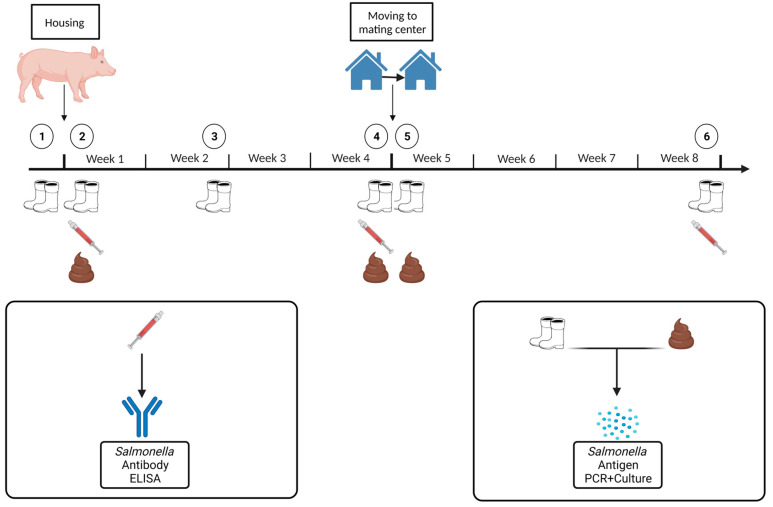
Scheme of gilt integration with sampling time points. (Created with BioRender.com, accessed on 17 January 2023).

In both parts of the study, a total of six groups of gilts per diet group were investigated on the farm. The detection of *Salmonella* by antigen in boot swabs (integration of gilts at six time points) was conducted and fecal samples (integration of gilts at three time points) were taken specifically at certain time points during the feeding of the two diets. *Salmonella* antibodies were measured in the animals’ serum (integration of gilts at three time points) (Figure 1).

#### 2.4.2. Peripartal Sows

For each part of the trial, four experimental and control groups of sows were investigated in the farrowing unit. The trial was interrupted in summer to avoid reduced feed intake at high temperatures. The sows were housed one week before weaning. During the housing period, one week before farrowing, boot swabs were taken in the clean pens. In addition, boot swabs were taken at the weaning of the piglets to record the *Salmonella* load at the end of the run. 

**Figure 2:**
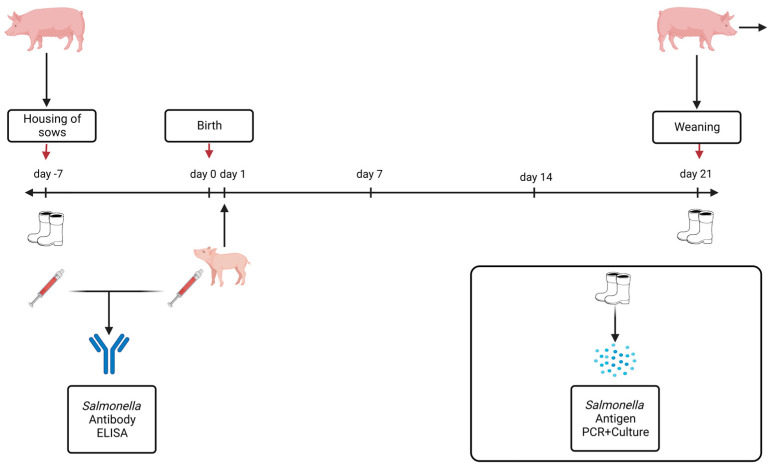
Scheme of investigation with sampling time points. (Created with BioRender.com, accessed on 17 January 2023).

Serum samples were also collected to determine the prevalence of *Salmonella* via *Salmonella* antibodies at the time of housing. In addition, serum samples were collected from three piglets per litter on the day after birth (Figure 2). The sampling schedule is shown in Figure 2. 

#### 2.4.3. Piglet Rearing

**Figure 3:**
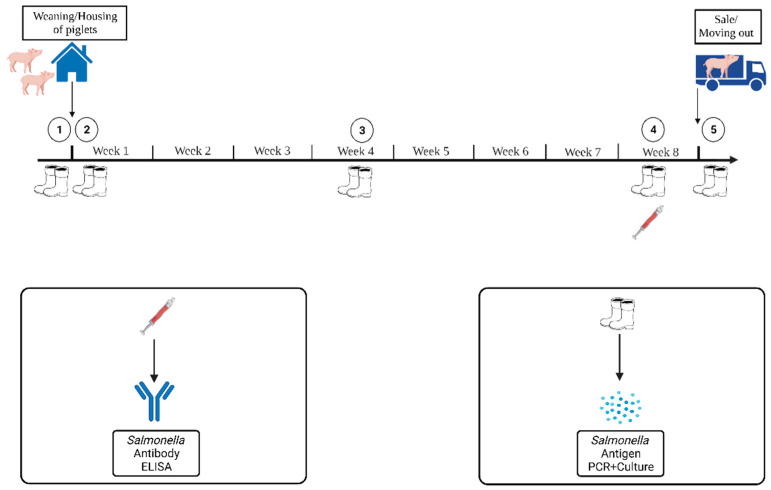
Scheme of investigation in the piglet rearing unit with sampling time points. (Created with BioRender.com, accessed on 17 January 2023).

A total of four runs were sampled in the piglet-rearing unit. Four runs of piglets on Farm A (n = 224 piglets) and B (n = 144 piglets) and six runs on Farm C (n = 228 piglets) were investigated. On the farm, piglets were randomly selected for sampling. Sampling was carried out according to the scheme shown in Figure 3. The sampling period started when the piglets were housed in the pen and ended when the animals were taken out of the pen or sold. For this purpose, five boot swabs were taken during this period, the first of which was taken in the cleaned and disinfected pen before the piglets were housed. To see a potential increase in *Salmonella* antibodies in the serum, the blood sample was taken only one week before housing. Fecal samples were not taken during piglet rearing. A total of 343, 196, and 301 sock swabs were taken on Farms A, B, and C, respectively. Furthermore, 225, 217, and 229 blood samples were taken on Farms A, B, and C. 

### 2.5. Collection of Samples

#### 2.5.1. Boot Swabs

Environmental boot swabs were taken to detect the *Salmonella* antigen. The swab (HygroStar, Franz Mensch GmbH, Buchloe, Germany) was pulled over a boot previously covered with a plastic overshoe (WDT, Garbsen, Germany). The pen was tested according to a standardized protocol to ensure the comparability of samples. First, the outer walls of the pen were walked along, then the pen was meandered through to sample as large an area as possible [26]. Boot swabs in gilt integration were taken at six time points in three runs per feeding group in Farm A (four pens), Farm B (one pen), and Farm C (four pens). This gives a total of n = 72 boot swabs in Farms A and C and n = 18 in Farm B in gilt integration per feeding group.

In the farrowing unit, every farrowing pen from the sows was tested. Boot swabs were collected from Farms A (n = 10 farrowing pens), B (n = 10 farrowing pens), and C (n = 9 farrowing pens) at two time points in four runs, resulting in a total of n = 80 (Farm A + B) or n = 72 (Farm C) boot swabs. 

In the piglet rearing, boot swabs were taken in Farm A (eight pens in four runs), Farm B (six pens in three runs), and Farm C (21 pens in one run) at five time points per feeding group. In Farm C, the third time point was split resulting in seven time points. Thus, in Farm A, n = 160; Farm B, n = 90; and Farm C, n = 147 boot swabs per feeding group were tested.

After sampling, the swabs were packaged and sent to the veterinary diagnostic laboratory (SAN Group Biotech Germany GmbH, Höltinghausen, Germany).

#### 2.5.2. Fecal Samples

Fecal samples were collected from spontaneously defecating animals using a clean glove. Approximately 10 g of feces were transferred into a sample container. The fecal samples were then sent to the veterinary diagnostic laboratory (SAN Group Biotech Germany GmbH).

#### 2.5.3. Blood Samples

Blood samples were taken from the external jugular vein of the sows and from the cranial vein of the piglets [29]. The blood was collected in a tube containing coagulation factor (Sarstedt Serum Monovette^®^, Sarstedt AG & Co. KG, Nümbrecht, Germany). The blood was centrifuged at 3000 rpm for 6 min and the obtained serum was sent to a veterinary diagnostic laboratory (SAN Group Biotech Germany GmbH).

### 2.6. Salmonella Detection

The study was conducted in collaboration with the veterinary diagnostic laboratory SAN Group Biotech Germany GmbH, Höltinghausen, Germany.

#### 2.6.1. Boot Swabs and Fecal Samples

*Salmonella* testing was performed using the KYLT^®^ PCR detection method (SAN Group Biotech Germany GmbH, test authorization FLI-B 656, sensitivity and specificity 100%). Environmental boot swabs, individual fecal samples, and feed samples were tested. SAN Group Biotech Germany GmbH carried out the tests. The samples were first enriched in peptone water and then analyzed by real-time PCR. Samples with positive PCR results were tested according to the Kauffman–White scheme by enrichment on modified Semi-Solid Rappaport-Vassiliadis (MSRV) agar [30]. After pre-enrichment, samples were cultured on Rambach and xylose-lysine-deoxycholate (XLD) selective media and examined macroscopically. Vaccine-specific *Salmonella* Typhimurium DIVA real-time PCR (Kylt^®^ ST DIVA, SAN Group Biotech Germany GmbH) was performed in one case to differentiate between field strains and the vaccine strain. Serotyping was performed by subculturing colonies on blood agar and a rapid slide agglutination test with sera (Sifin Diagnostics GmbH, Berlin, Germany) to determine surface antigens [31].

#### 2.6.2. Blood Samples

In addition, an indirect detection method for serum samples was used to detect antibodies against lipopolysaccharide of *Salmonella* serovars of groups B, C, D, and E. The pigtype *Salmonella* Ab ELISA, version May 2010 (Indical Bioscience GmbH, Leipzig, Germany) was used.

### 2.7. Statistical Analysis

SAS Enterprise Guide (version 7.1, SAS Institute Inc., Cary, NC, USA) was used for statistical analysis. Differences in the distribution of positive and negative *Salmonella* samples from each diet group were analyzed at the farm level using the chi-squared homogeneity test. The chi-square homogeneity test differentiates the sample result distribution for each time point individually. Based on the distributions of the OD values (optical density), the Wilcoxon rank sum test was chosen. In the Wilcoxon rank sum test, the two feeding groups (control and experimental) were tested for significant differences for each farm separately in the area of gilt integration, farrowing unit, and piglet rearing unit. Differences with a significant level of *p* < 0.05 indicate statistically different frequencies of positive samples according to sample type [32].

## 3. Results

### 3.1. Salmonella Prevalence

On all three farms, positive *Salmonella* samples were found in the respective subunits. In the following, the results of the study are presented at the farm level, since a comparison between farms is not meaningful.

#### 3.1.1. Farm A

The results of the *Salmonella* prevalence on Farm A, divided according to the feeding groups and the age of the animals, are shown in Table 7. The data were based on a total of *n* = 624 boot swabs, *n* = 90 fecal samples, and *n* = 778 serum samples in the first part of the trial (rye) and *n* = 304 boot swabs, *n* = 90 fecal samples and *n* = 234 serum samples in the second part of the trial (rye bran). A similar number of positive boot swabs were found when comparing the diet groups during the gilt integration. Positive fecal samples were found in only one run in the rye bran group (6 of 45). The average OD values behaved similarly to the number of positive boot swabs. In the experimental groups, the mean OD rose later than in the control groups. 

In the farrowing unit, no positive swabs were found when the sows were housed. At the end of lactation, only one positive swab was found in the rye group in the first part of the trial. In the second part of the trial, there were significantly more positive swabs, with 9 positive swabs in the control group and 11 in the experimental group. 

Feeding in piglet rearing was only carried out in the first part of the trial, so data were only available from two feeding groups. Here, more positive *Salmonella* swabs were found in the rye group than in the control group (88 vs. 71).

When comparing the *Salmonella* swabs in the farrowing unit, it is noticeable that only one positive swab was found in the first (test and control) runs. Looking at the rye bran runs, which were collected at different times, it is noticeable that also no positive swab could be found at stabling. 

#### 3.1.2. Farm B 

The results of the *Salmonella* prevalence on Farm B according to the feeding groups and the age of the animals are shown in Table 8. The data are based on a total of *n* = 374 boot swabs, *n* = 86 fecal samples, and *n* = 522 serum samples in the first part of the experiment (rye). Feeding rye bran was not evaluated on Farm B. 

When comparing the feeding groups in gilt integration, there were only a few positive samples. In total, only two of 18 positive swabs were found during one experimental run, while in the control group, all swabs were negative. A comparison of the fecal samples showed a similar pattern. Again, only three of 42 positive fecal samples were found in the experimental group. A comparison of the OD values showed similar values between the feeding groups. 

In the peripartum period, only a few positive *Salmonella* boot swabs were found in the clean farrowing pens of both feeding groups prior to housing. At weaning, only one positive swab was found in the experimental group. The OD values of sows and piglets were also similar between the two feeding groups. 

In the piglet rearing area, a similar number of positive swabs was found between the feeding groups at the respective times. This was also reflected in the mean OD values before moving out (Control = 53.1 vs. Experimental = 47.1). 

#### 3.1.3. Farm C

The results of *Salmonella* prevalence on Farm C according to feeding groups and age of the animals are shown in Table 9. The data were based on a total of n = 584 boot swabs, n = 180 fecal samples, and n = 235 serum samples in the first part of the trial (rye). Feeding rye bran was also not evaluated on Farm C. When comparing the boot swabs taken during the integration of the gilts, it is noticeable that positive *Salmonella* swabs were found in the control group at the time of first housing. In the experimental group, positive swabs were only found after transfer to the mating center. No positive swabs were found in this group prior to rehousing. The fecal samples showed a similar picture. Positive samples were found in the control group prior to rehousing and in the rye group after rehousing. The OD values of the serum samples increased more in the control group than in the rye group (at the end: Control = 109.3 vs. Experimental = 69.0).

In the farrowing unit, positive swabs were found in both feeding groups even during sampling in the clean barn. At the time of weaning, a similar number of positive swabs from both feeding groups were found. The OD values of the serum samples were almost identical in both groups for pre-housing.

In the piglet rearing area, fewer positive swabs were found on Farm C. A comparable number of positive swabs were found at the time of first stabling. In the control group, positive swabs were found sporadically at different times, whereas in the rye group, no positive swab was found after the time of first stabling. The OD values were at a very low comparable level in both feeding groups. When comparing the number of positive samples for all ages, a significantly lower positive number was found in the experimental group on Farm C samples (boot swabs: 53 vs. 22 and feces: 10 vs. 3).

### 3.2. Salmonella OD Values

#### 3.2.1. Rye

When comparing the OD values of the different feeding groups on the farms, it is noticeable that the OD values of the gilts on Farm A were higher from the second time point onwards. When comparing the two diet groups, clear differences can be seen. In gilt integration, the OD values of the rye group were significantly lower than those of the control group at time points 2 and 3 on Farms A (85.47 and 116, respectively) and C (20.41 and 68.98, respectively), whereas there was no difference in the OD values on Farm B. In the peripartal period, no significant differences were found between the OD values. In the piglet rearing unit, the OD values of the rye group on Farms A and B were significantly lower than those of the control group. On Farm C, the very low OD values were not different. The results can be seen in Table 10.

#### 3.2.2. Rye Bran

When comparing the OD values of the second control group with the experimental group (rye bran), no difference between the OD values of the gilts was noticeable. The *Salmonella* OD value of the sows was also almost identical. Since the feeding concept with rye bran was not applied in piglet rearing, no data were available for this. The results can be seen in Table 11.

### 3.3. Salmonella Serovars of PCR-Positive Samples

**Figure 4:**
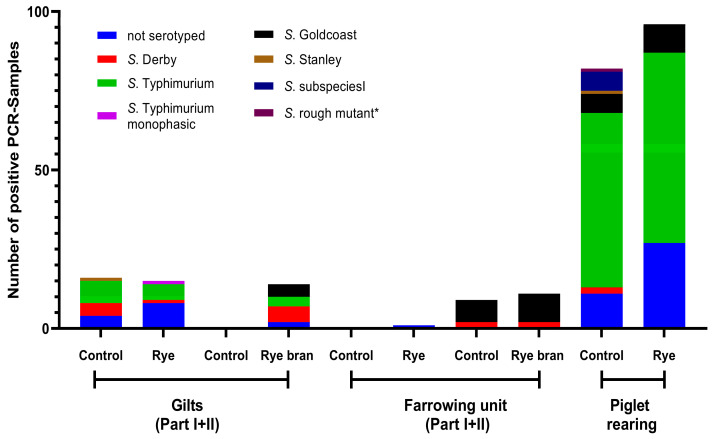
Number of positive PCR samples of boot swabs and feces of Farm A and proportionally their cultivation and serovars. * Mutant of *S. typhimurium* with altered lipopolysaccharide O-antigen.

**Figure 5:**
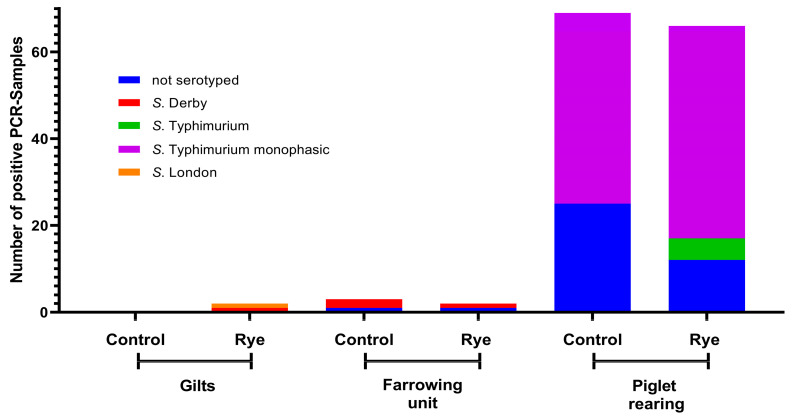
Number of positive PCR samples of boot swabs and feces of Farm B and proportionally their cultivation and serovars.

**Figure 6:**
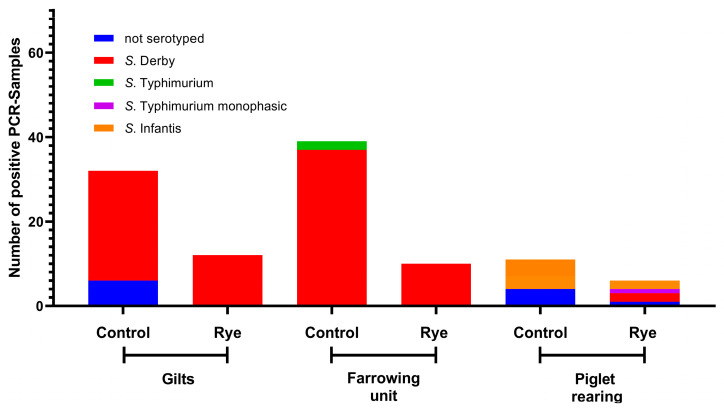
Number of positive PCR samples of boot swabs and feces of Farm C and proportionally their cultivation and serovars.

On all farms (A, B, and C), a total of *n* = 498 positive samples were found, of which 393 were serotyped (78.91%). The number of positive PCR samples per farm and, if available, their serotyping are shown in Figure 4, Figure 5 and Figure 6.

## 4. Discussion

In recent years, rye has become increasingly popular as a feed grain [33]. Through breeding efforts, the risk of ergot contamination is low in some varieties, so rye can also be used in breeding animals without the risk of ergot contamination, particularly in pig production [10]. In addition, rye is also an attractive cereal in terms of sustainability. It requires less fertilizer and water than wheat and can reduce CO_2_ emissions [9]. Moreover, the use of rye by-products from the food industry is of course even better in terms of sustainability [22]. Rye and rye bran have a high content of digestible dietary fiber, the NSP [14]. Microorganisms are able to convert these NSPs into volatile fatty acids in the large intestine, producing, besides other SCFA, the volatile fatty acid butyrate [34]. Butyrate in the large intestine strengthens the epithelial barrier function and inhibits the spread of *Salmonella* in the intestinal tract [17,18]. In the present study, the influence of rye and rye bran on *Salmonella* prevalence in piglet production was evaluated.

### 4.1. Epidemiological Situation

On all three farms, the *Salmonella* problems mentioned in the pre-selection were observed and *Salmonella* was found in all age groups. Several cohorts were examined over a period of several months in order to exclude possible fluctuations and influences [34]. 

Biosecurity is an essential point in the control of *Salmonella* on pig farms, for which optimal cleaning and disinfection are essential to avoid reinfection of the animals [4]. On all three farms, few positive samples were found in the cleaned and disinfected barns, so there is also a possibility for improvement here. *Salmonella* in the clean barn was detected in the boot swabs of gilts (Farm A; *n* = 1 of 48), farrowing pens (Farm B, *n* = 4 of 80; Farm C *n* = 3 of 72) and piglet rearing pens (Farm A, *n* = 31 of 160; Farm B, *n* = 16 of 88).

To allow for discussion, the results of all farms were discussed in age groups independent of the farm.

#### 4.1.1. Influence of Rye and Rye Bran on Salmonella Prevalence in Gilt Integration

Gilt integration is a critical point in terms of *Salmonella* infection [27]. When comparing the number of positive boot swabs, no difference could be found between the feeding groups. When comparing the *Salmonella* OD values between the feeding groups, it is noticeable that on Farm A (vaccinated) and Farm C (non-vaccinated), the *Salmonella* OD values at time points two and three were significantly lower with a rye-rich diet. Farm A actually had high OD values. This can be explained by the double vaccination with an attenuated *S. typhimurium* vaccine strain (Salmoporc^®^, Ceva Tiergesundheit GmbH). When looking at the *Salmonella* serotypes in Figure 4, it is noticeable that in Farm A, *S. typhimurium* was clearly a more frequent serotype. Nevertheless, various other serotypes were also found, which indicates an infection event. Due to the vaccination on Farm A, it can therefore be assumed that more positive environmental samples are to be found as described by Buch, et al. [35]. Nevertheless, they reported that vaccination alone cannot influence *Salmonella* prevalence on farms [35]. 

The optical density of serum samples is directly related to the amount of *Salmonella* antibodies [36]. Hollmann, et al. [26] described that interpreting serum samples alone leads to potentially erroneous results because there is a correlation between age and the level of antibody titer. Indeed, Wilhelm, et al. [37] showed that the older an animal is, the more likely it is to find high OD values. However, as gilts of the same age were used in our study, these OD values are comparable. When looking at the number of positive boot swabs, it was noticeable that, in the experimental groups (rye and rye bran), they increased much later than in the control group. It can be assumed that a diet rich in rye and rye bran in the gilts may lead to a delayed infection, but not to a reduced number of positive samples overall. 

The total number of positive fecal samples was very low. Chuppava, et al. [20] showed that the use of rye in the diet can lead to reduced fecal shedding of *Salmonella*. In addition, Hankel, et al. [38] have shown that feeding high levels of rye instead of wheat promotes the growth of beneficial gut bacteria while reducing the growth conditions for *Salmonella* Typhimurium. However, both of the above studies took place under standardized infection trials. In addition, these studies worked with much higher proportions of rye in the diet. In our study, no effect on *Salmonella* shedding was observed due to the low number of positive fecal samples in both feeding groups. Because *Salmonella* is not continuously excreted, fecal samples are not very reliable in providing an accurate picture of *Salmonella* prevalence [39,40]. Contrary to what is described in the literature [20,38], no comparable effect of rye on *Salmonella* in the boot swabs and fecal samples could be seen based on the investigations in this study under field conditions.

Based on the lower OD values in the serum samples in our study in the rye group with a similar infection pattern, an effect of rye on *Salmonella* antibodies can be described. This effect on OD values could not be demonstrated when rye bran was fed. 

#### 4.1.2. Influence of Rye and Rye Bran on Salmonella Prevalence in the Farrowing Unit

In our study, the farrowing unit had the fewest overall positive boot swabs and fecal samples. In the comparison of the positive samples found, no difference between the feeding groups could be shown. The OD values of the sows in the farrowing unit were also not significantly different. During the farrowing period, sows are exposed to particular stress [41]. It is described in the literature that, especially during stress, sows are more susceptible to infectious diseases and carrier sows are more likely to excrete *Salmonella* [42]. This effect could not be shown in our study. The transfer of *Salmonella* antibodies to the suckling piglets is an important part of *Salmonella* prevention [43]. When comparing the blood samples of the sows with the 24 h old piglets, an adequate colostrum supply, via the transfer of *Salmonella* antibodies, could be found on all three farms and in all feeding groups. This adequate colostrum supply can possibly reduce *Salmonella* prevalence in the subsequent piglet rearing [43]. However, the positive swabs in the farrowing unit did not show any differences between the feeding groups, so additionally there was no improvement in colostrum supply via the diet. 

#### 4.1.3. Influence of Rye on Salmonella Prevalence in Piglet Rearing

The literature describes that the highest number of positive *Salmonella* samples are found in piglet rearing [26,35,44,45]. This is explained by the drop in maternal antibodies between the fourth and eighth week of life so the piglets are more susceptible to *Salmonella* infection [27,46]. This hypothesis was also shown on Farm A and Farm B. On Farm C, however, most positive *Salmonella* samples were found in the gilts.

Referring to the results of Chuppava, et al. [20], positive effects of rye in piglet rearing had been expected here. However, when comparing positive boot swabs, no difference was found between the feeding groups. Nonetheless, there was an effect of feeding on the OD values similar to that observed in the gilts. With almost the same number of positive swabs in the piglet-rearing group, a significantly lower OD value was found at the time of blood sampling on Farm A and Farm B.

### 4.2. Influence of Rye and Rye Bran on Salmonella Serovars

The gilts and sows could be an important source of *Salmonella* persistence on pig farms [47]. The consideration of the serovars is not unimportant, since there are several studies that indicate, that duration of *Salmonella* excretion is directly related to the serovar, as well as the survival time of some *Salmonella* serovars [48,49]. The prevalence of serovars in the different age groups provides information on the extent to which serovars from gilts and sows are also found in the piglet-rearing area. The serovars most commonly found in our study, *Salmonella* Typhimurium, *Salmonella* Derby, and the monophasic variant of *Salmonella* Typhimurium are consistent with the literature of the most commonly found serovars in the EU [50]. Nevertheless, other serovars such as *Salmonella* Goldcoast or *Salmonella* Infantis were also found in our study. Although in much smaller proportions. The serovars of the different age groups can be classified differently depending on the farm. On Farm A, almost the same serovars were found in the sows and in the piglets, a phenomenon that was also reported in the literature [45]. On Farms B and C different serovars were found in addition to the similar serovars. The correlation of serovars in gilts, sows, and piglet rearing is therefore not clearly given and is also farm specific.

## 5. Conclusions

Rye and rye bran as a feed component for gilts, sows, and in piglet rearing did not show a clear impact on *Salmonella* prevalence in our study. The analysis of the sock swabs and also the fecal samples showed no effect on *Salmonella* prevalence, while serum samples showed an effect on OD values, which alone could not be clearly attributed to the effects of the rye or rye bran. However, the use in gilt integration, sow management, and piglet rearing did not demonstrate any negative effects either. By combining the detection methods used to determine *Salmonella* prevalence, a non-constant *Salmonella* incidence was detected in the different areas. Thus, a possible effect of rye as well as rye bran was difficult to prove under these conditions. Further studies with a higher number of animals and a longer time period are necessary to show a clear effect of both these ingredients.

## Figures and Tables

**Table 1:** Percentage of rye and rye bran in the experimental diets in all farms.

	Rye	Rye Bran
Gilts	30%	20%
Sows	30%	15%
Piglet rearing	25%	-

**Table 2:** Composition of the control as well as the experimental diets for gilts (rye).

	Farm A		Farm B		Farm C	
Ingredients %	CON 1	RYE	CON	RYE	CON	RYE
Wheat	10.0	-	17.0	5.0	5.0	5.0
Barley	53.0	19.5	45.0	24.5	50.0	16.1
**Rye**	**-**	**30.0**	**-**	**30.0**	**-**	**30.0**
Wheat bran	10.0	22.4	14.0	12.5	-	-
Wheat semolina bran	-	-	8.5	9.0	-	-
Cereal bran	-	-			25.0	25.0
Sugar beet pulp, molassed	1.4	2.8	4.0	4.5	2.9	5.0
Sugar beet molasses	-	-	0.6	-	-	1.5
Soybeans, toasted	-	-	2.5	-	-	-
Soybean extraction meal	14.5	16.4	1.5	8.2	8.0	8.0
Sunflower extruded meal	-	-	4.0	2.1	2.0	2.0
Rapeseed extraction meal	3.0	-	-	-	3.0	3.2
Rapeseed oil	0.6	1.1	-	-	-	-
Malt germs	5.0	5.0	-	-	-	-
Lignocellulose	-	-	-	1.0	-	-
Calcium carbonate	0.9	1.1	1.2	1.1	1.1	0.7
Other ingredients	1.6	1.7	1.7	2.1	3.0	3.5

Values have been rounded.

**Table 3:** Composition of the control as well as the experimental diets for gilts (rye bran).

	Farm A	
Ingredients %	CON	RB
Wheat	10	-
Barley	53	51.7
**Rye bran**	-	**20**
Wheat bran	10	-
Sugar beet pulp, molassed	1.5	2.7
Soybean extraction meal	14.5	13.6
Rapeseed extraction meal	3	3
Malt germs	5	6
Calcium carbonate	0.9	1.3
Other ingredients	2.1	1.7

Values have been rounded.

**Table 4:** Composition of the control as well as the experimental diets for lactating sows in the first part of the study (rye).

	Farm A		Farm B		Farm C	
Ingredients %	CON	RYE	CON	RYE	CON	RYE
Wheat	25	10	20.5	10	20	12.5
Barley	30	3.1	40	20	44.5	16.7
**Rye**	-	**30**		**30**	-	**30**
Wheat bran	9	16.4	1.6	1.6	-	-
Wheat semolina bran	-	-	13.5	9.4	-	-
Wheat gluten feed	-	-	-	-	9.6	10.1
Cereal bran	-	-	-	-	3.4	8.6
Corn	9	10	-	-	-	-
Sugar beet pulp, molassed	-	-	2.8	3	2	2
Soybeans, toasted	-	5	4.5	5	-	-
Soybean extraction meal	12	9.2	10	13	14	14.7
Rapeseed extraction meal	9	10	-	-	-	-
Rapeseed oil	2.3	2.3	-	-	-	-
Soybean oil	-	-	-	-	1.2	1
Linseed	1	0.6	2.7	2.7	-	-
Calcium carbonate	1	0.9	1.2	1.2	1.4	1.4
Other ingredients	1.7	2.5	3.2	4.1	3.9	3

Values have been rounded.

**Table 5:** Composition of the control as well as the experimental diets for lactating sows in the second part of the study (rye bran).

	Farm A			
KoIngredients %	CON_1_	RB_1_	CON_2_	RB_2_
Wheat	25	20	36.00	36
Barley	30	25.8	30.00	25
**Rye bran**	**-**	**15**	**-**	**15**
Wheat bran	9	-	8.2	-
Corn	9	6.9	-	-
Sugar beet pulp, molassed	-	2.5	2.00	2
Soybeans, toasted	-	1.7	-	-
Soybean extraction meal	12	12	12.7	12
Raps extraction meal	9	9.7	1.00	1
Rapeseed oil	2.5	2.5	-	0.3
Linseed	1	0.6	1.40	1.3
Calcium carbonate	1	0.6	0.8	1.6
Baking and pastry industry (wafer flour)	-	-	5.80	4
Other ingredients	1.5	3.3	2.1	3.4

Values have been rounded; x_1_ diets in runs one and two; x_2_ diets in runs three and four.

**Table 6:** Composition of the control as well as the experimental diets for piglet-rearing units.

Ko	Farm A		Farm B		Farm C			
KoIngredients (% uS)	CON	RYE	CON	RYE	CON_1_	RYE_1_	CON_2_	RYE_2_
Wheat	34	20.5	24	2.4	34	20.5	40.5	15.5
**Rye**		**25**		**25**		**25**		**25**
Barley	38.5	23.5	20	20	38.5	23.5	29	26.5
Barley (digested)	-	-	8	6	-	-	-	-
Wheat bran	2.7	2.5	-	-	2.7	2.5	3.5	3.5
Wheat semolina bran	-	-	5	3.3	-	-	-	-
Corn	-	-	-	-	-	-	5	5
Corn (digested)	-	-	8	6	-	-	-	-
Bread flour	-	-	6	5	-	-	-	-
Bakery by-products	-	-	2.5	2.1	-	-	-	-
Soybeans heated	2.2	2.5			2.2	2.5	1.3	1.3
Soybean extraction meal	14.2	16	10	18.1	14.2	16	14.5	17
Soybeans toasted			8	3	-	-	-	-
Soybean hulls	0.7	1.4	-	-	0.7	1.4	1.3	1.3
Linseed meal(partially extracted)	1.6	2.5	-	-	1.6	2.5	0.8	0.8
Soybean oil	2	2.1	1	2	2	2.1	1	1
Sucrose (beet sugar)	-	-	1.2	1.2	-	-	-	-
Other ingredients	4.1	4	6.3	5.9	4.1	4	3.1	3.1

Values have been rounded; x_1_ Diets in the third phase of piglet rearing; x_2_ Diets in the fourth phase of piglet rearing.

**Table 7:** Comparison of *Salmonella* positive results of Farm A for the different feeding groups divided according to gilt integration, peripartal, and piglet rearing.

Time Point			Control I			Experimental I (Rye)			Control II			Experimental II (Rye Bran)		
			Pos. Samples/%		Average OD %	Pos. Samples/%		Average OD %	Pos. Samples/%		Average OD %	Pos. Samples/%		Average OD %
No.	Week	Event	Bs	f		Bs	f		Bs	f		Bs	f	
			Gilt integration											
		*n*	72	45	117	72	45	117	72	45	117	72	45	117
1	−1	Pre-housing	0/0.0	-	-	1/8.3	-	-	0/0.0	-	-	0/0.0	-	-
2	0	Housing	4/33.3	0/0.0	21.98	1/8.3	0/0.0	17.5	2/16.7	0/0.0	12.3	0/0.0	0/0.0	15.9
3	2	Half of quar.	7/58.3	-	-	1/8.3	-	-	0/0.0	-	-	0/0.0	-	-
4	4	End of quar.	5/41.7	0/0.0	148.84	6/50.0	0/0.0	85.5	0/0.0	0/0.0	65.8	5/41.2	3/20.0	64.2
5	5	Moving to m.c.	0/0.0	0/0.0	-	2/16.6	0/0.0	-	0/0.0	0/0.0	-	5/41.2	3/20.0	-
6	8	End	0/0.0	-	147.01	4/33.3	-	116.1	0/0.0	-	86.0	4/33.3	-	97.0
		Total gilts	16/22.2	0/0.0	-	15/20.8	0/0.0	-	2/2.8	0/0.0	-	14/19.4	6/13.3	-
			Peripartal											
		*n*	80	-	160	80	-	160	80	-	160	80	-	160
1	−1	Pre-housing	0/0.0	-	55.0	0/0.0	-	61.8	0/0.0	-	62.0	0/0.0	-	64.7
2	1	Farrowing (piglets)	-	-	44.7	-	-	50.91	-	-	52.8	-	-	49.2
3	3	Weaning	0/0.0	-	-	1/2.5	-	-	9/22.5	-	-	11/27.5	-	-
		Total peripartal	0/0.0	-	-	1/1.3	-	-	9/11.3	-	-	11/13.8	-	-
			Piglet rearing											
		*n*	160		112	160		112	-	-	-	-	-	-
1	−1	Pre-housing	13/40.1	-	-	18/56.3	-	-	-	-	-	-	-	-
2	0	Housing	9/28.1	-	-	8/25.0	-	-	-	-	-	-	-	-
3	3	Midpoint	18/56.3	-	-	26/81.3	-	-	-	-	-	-	-	-
4	7	Before moving out	19/59.4	-	25.3	33/51.6	-	14.7	-	-	-	-	-	-
5	8	After moving out	12/37.5	-	-	14/43.8	-	-	-	-	-	-	-	-
		Total piglets	71/44.4	-	-	88/55.0	-	-	-	-	-	-	-	-
*n*			312	45		312	45		152	45		152	45	
Total farm A			87/27.9	0/0.0		108/33.7	0/0.0		11/7.2	0/0.0		25/16.4	6/13.3	

Event: quar. = quarantine, m.c. = mating center; bs = boot swab, f = fecal; OD = optical density, “-” not applicable.

**Table 8:** Comparison of *Salmonella* positive results of Farm B for the different feeding groups divided according to gilt integration, peripartal, and piglet rearing.

Time Point			Control			Experimental Rye		
			Pos. Samples/%		Average OD %	Pos. Samples/%		Average OD %
No.	Week	Event	Bs	f		Bs	f	
			Gilt integration					
		*n*	18	44	102	18	42	105
1	−1	Pre-housing	0/0.0	-	-	0/0.0	-	-
2	0	Housing	0/0.0	0/0.0	37.2	1/33.3	1/6.7	33.0
3	2	Half of quar.	0/0.0	-	-	1/33.3	-	-
4	4	End of quar.	0/0.0	0/0.0	47.6	0/0.0	2/13.3	48.1
5	5	Moving to m.c.	0/0.0	0/0.0	-	0/0.0	0/0.0	-
6	8	End	0/0.0	-	35.4	0/0.0	-	45.4
		Total gilts	0/0.0	0/0.0	-	2/11.1	3/7.1	-
			Peripartal					
		*n*	80	-	157	80	-	157
1	−1	Pre-housing	3/7.5	-	41.2	1/2.5	-	39.0
2	1	Farrowing	-	-	22.8	-	-	26.33
3	4	Weaning	0/0.0	-	-	1/2.5	-	-
		Total peripartal	3/3.8	-	-	2/2.5	-	-
			Piglet rearing					
		*n*	88	-	108	90	-	108
1	−1	Pre-housing	5/27.8	-	-	11/61.1	-	-
2	0	Housing	9/56.3	-	-	8/44.4	-	-
3	4	Midpoint	16/88.9	-	-	13/72.2	-	-
4	7	Before moving out	18/100.0	-	53.1	15/83.3	-	47.1
5	8	After moving out	13/72.3	-	-	12/66.7	-	-
		Total piglets	61/69.3	-	-	59/65.6	-	-
*n*			186	44		188	42	
Total farm B			64/34.4	0/0.0		63/26.3	38,414	

Event: quar. = quarantine, m.c. = mating center; bs = boot swab, f = fecal; OD = optical density,; “-” not applicable.

**Table 9:** Comparison of *Salmonella* positive results of Farm C for the different feeding groups divided according to gilt integration, peripartal, and piglet rearing.

Time Point			Control			Experimental Rye		
			Pos. Samples/%		Average OD %	Pos. Samples/%		Average OD %
No.	Week	Event	Bs	f		Bs	f	
			**Gilt integration**					
		*n*	72	90	117	72	90	117
1	−1	Pre-housing	0/0.0	-	-	0/0.0	-	-
2	0	Housing	4/33.3	0/0.0	25.7	0/0.0	0/0.0	22.1
3	2	Half of quar.	4/33.3	-	-	0/0.0	-	-
4	4	End of quar.	9/75.0	5/33.3	55.9	0/0.0	0/0.0	20.4
5	5	Moving to m.c.	10/83.3	5/33.3	-	8/66.7	-	-
6	8	End	5/41.6	-	109.3	4/33.3	3/20.0	69.0
		Total gilts	32/44.4	10/22.2	-	12/16.7	3/6.7	-
			Peripartal					
		*n*	72	-	195	72		197
1	−1	Pre-housing	1/2.6	-	80.0	2/5.1	-	80.3
2	1	Farrowing	-	-	75.1	-	-	59.95
3	4	Weaning	9/23.1	-	-	8/20.5	-	-
		Total peripartal	10/12.8	-	-	10/12.8	-	-
			Piglet rearing					
		*n*	148	-	115	148		114
1	−1	Pre-housing 1	0/0.0	-	-	0/0.0	-	-
2	0	Housing 1	5/23.8	-	-	6/28.6	-	-
3	3	Midpoint	2/9.5	-	-	0/0.0	-	-
3a	3	Pre-housing 2	0/0.0	-	-	0/0.0	-	-
3b	4	Housing 2	3/14.2	-	-	0/0.0	-	-
4	6	Before moving out	1/4.8	-	4.8	0/0.0	-	2.3
5	7	After moving out	0/0.0	-	-	0/0.0	-	-
		Total piglets	11/7.4	-	-	6/4.1	-	-
*n*			292	90		292	90	
Total farm C			53/18.2	10/22.2		22/7.5	3/6.7	

Event: quar. = quarantine, m.c. = mating center; bs = boot swab, f = fecal; OD = optical density; “-” not applicable.

**Table 10:** Comparison of *Salmonella* OD Value (%) results for control and rye sows divided according to gilt integration, peripartal, and piglet rearing.

Timepoint	Farm A				Farm B				Farm C			
	n	Control	Rye	*p*-Value	n	Control	Rye	*p*-Value	n	Control	Rye	*p*-Value
**Gilts**												
1	39/39	21.98 ± 17.6	17.46 ± 14.0	0.2363	34/35	37.21 ± 37.6	33.03 ± 20.9	0.6383	39/39	25.72 ± 24.0	22.1 ± 15.1	0.8260
2	39/39	148.8 ± 28.8	85.47 ± 38.7	**<0.0001**	34/35	47.61 ± 29.4	48.07 ± 29.9	0.8010	39/39	55.91 ± 51.1	20.41 ± 26.3	**0.0002**
3	39/39	147.0 ± 32.2	116.1 ± 48.1	**0.0003**	34/35	35.37 ± 25.0	45.38 ± 29.7	0.1657	39/39	109.27 ± 40.7	68.98 ± 40.7	**<0.0001**
**Peripartal**												
1	40/40	55.0 ± 25.9	61.8 ± 31.3	0.4217	40/40	41.16 ± 36.7	38.98 ± 25.0	0.6790	72/74	79.98 ± 41.6	80.28 ± 36.6	0.8726
**Piglet rearing**												
1	112/112	25.3 ± 31.1	14.72 ± 19.2	**0.0003**	108/108	53.09 ± 41.6	47.06 ± 40.2	**0.0358**	115/114	4.83 ± 18.0	2.32 ± 4.7	0.1452

C = Control group R = Experimental group (Rye); %. *p*-value of *t*-test homogeneity test < 0.05 was considered significant (bold).

**Table 11:** Comparison of *Salmonella* OD Value (%) results for control 2 and rye bran divided according to gilt integration, peripartal, and piglet rearing.

Time Point	Farm A		
	Control (Part II)	Rye Bran	*p*-Value
**Gilts**			
1	12.33 ± 12.47	15.90 ± 16.0	0.2133
2	65.84 ± 34.5	64.25 ± 34.5	0.9482
3	86.03 ± 24.3	97.00 ± 53.1	0.0795
**Peripartal**			
1	62.02 ± 34.7	64.66 ± 43.8	0.8852
**Piglet rearing**			
1	-	-	-

## Data Availability

The data presented in this study are available on request from the corresponding author.

## Supplementary Materials

The following supporting information can be downloaded at: https://www.mdpi.com/article/10.3390/ani13142262/s1, Table S1. Nutrient composition of compound feeds at farm A (g/kg as fed). Table S2. Nutrient composition of compound feeds at farm A (rye bran) (g/kg as fed). Table S3. Nutrient composition of compound feeds at farm B (g/kg as fed). Table S4. Nutrient composition of compound feeds at farm C (g/kg as fed).
