# Supplementary material for: Rye and Rye Bran as Components of Diets in Piglet Production—Effects on Salmonella Prevalence
Source: Animals (Basel). 2023 Jul 10;13(14):2262. doi: 10.3390/ani13142262 (PMC10376390; doi:10.3390/ani13142262)
Supplement: Supplementary file 1 [file animals-13-02262-s001.zip › animals-2471392-supplementary.pdf]

Table S1. Nutrient composition of compound feeds at farm A (g/kg as fed).

**Energy and nutritional content (g/kg)**	**Gilt integration**		**Farrowing unit**		**Piglet rearing**	
	Control	Rye	Control	Rye	Control	Rye
MJ (ME/kg as fed)]	12.6	12.6	13.4	13.4	13.5	13.5
Crude Protein [g/kg]	17.0	17.0	17.5	17.5	16.8	16.8
Crude Fat [g/kg]	3.0	3.5	5.7	6.0	4.5	4.6
Crude Fiber[g/kg]	5.8	5.8	5.0	5.0	4.0	4.0
Crude Ash [g/kg]	5.8	5.9	6.0	5.7	4.7	4.7
Ca [g/kg]	0.8	0.7	0.85	0.85	0.69	0.69
P [g/kg]	0.6	0.53	0.65	0.65	0.53	0.53
Na [g/kg]	0.25	0.25	0.25	0.25	0.23	0.23
Lys [g/kg]	0.95	0.95	1.05	1.05	1.27	1.27
Met [g/kg]	0.29	0.25	0.35	0.35	0.43	0.43

Table S2. Nutrient composition of compound feeds at farm A (rye bran) (g/kg as fed).

**Energy and nutritional content (g/kg)**	**Gilt integration**		**Farrowing unit**	
	Control	Rye bran	Control	Rye bran
MJ (ME/kg as fed)]	12.6	12.6	13.4	13.4
Crude Protein [g/kg]	17	17	17.5	17.5
Crude Fat [g/kg]	3.0	3.0	5.7	5.7
Crude Fiber[g/kg]	5.8	5.8	5.0	5.0
Crude Ash [g/kg]	5.8	5.9	6.0	5.8
Ca [g/kg]	0.8	0.7	0.85	0.85
P [g/kg]	0.6	0.53	0.65	0.65
Na [g/kg]	0.25	0.25	0.25	0.25
Lys [g/kg]	0.95	0.95	1.05	1.05
Met [g/kg]	0.29	0.25	0.35	0.35

Table S3. Nutrient composition of compound feeds at farm B (g/kg as fed).

**Energy and nutritional content (g/kg)**	**Gilt integration**		**Farrowing unit**		**Piglet rearing**	
	Control	Rye	Control	Rye	Control	Rye
MJ (ME/kg as fed)]	12.2	12.1	13.0	13.0	13.7	13.6
Crude Protein [g/kg]	14.0	14.0	16.5	16.5	17.0	17.0
Crude Fat [g/kg]	3.4	3.2	4.5	4.1	5.0	5.0
Crude Fiber[g/kg]	7.0	7.0	5.50	4.9	3.8	3.8
Crude Ash [g/kg]	5.0	5.0	5.6	5.7	4.7	5.0
Ca [g/kg]	0.7	0.68	0.9	0.9	0.7	0.7
P [g/kg]	0.55	0.53	0.65	0.65	0.55	0.55
Na [g/kg]	0.25	0.21	0.25	0.25	0.25	0.25
Lys [g/kg]	0.75	0.72	1.0	1.0	1.35	1.35
Met [g/kg]	0.24	0.21	0.3	0.3	0.43	0.42

Table S4. Nutrient composition of compound feeds at farm C (g/kg as fed).

**Energy and nutritional content (g/kg)**	**Gilt integration**		**Farrowing unit**		**Piglet rearing 1**		**Piglet rearing 2**	
	Control	Rye	Control	Rye	Control	Rye	Control	Rye
MJ (ME/kg as fed)]	11.9	11.9	13.2	13.2	13.5	13.5	13.4	13.4
Crude Protein [g/kg]	16	16	17	17	16.8	16.8	16.4	16.4
Crude Fat [g/kg]	3.7	3.1	3.9	4	4.0	4.6	3.4	3.4
Crude Fiber[g/kg]	6.8	6.2	4.9	4.9	4.0	4.0	4.1	4.1
Crude Ash [g/kg]	5.5	5.8	5.7	5.8	4.7	4.7	4.6	4.6
Ca [g/kg]	0.7	0.7	0.85	0.85	0.69	0.69	0.7	0.7
P [g/kg]	0.55	0.55	0.55	0.55	0.53	0.53	0.47	0.47
Na [g/kg]	0.2	0.2	0.25	0.25	0.23	0.23	0.23	0.23
Lys [g/kg]	0.8	0.8	0.95	0.95	1.27	1.27	1.15	1.15
Met [g/kg]	0.28	0.28	0.32	0.32	0.43	0.43	0.36	0.36
